# Safety Evaluation of a New Traditional Chinese Medical Formula, Ciji-Hua'ai-Baosheng II Formula, in Adult Rodent Models

**DOI:** 10.1155/2019/3659890

**Published:** 2019-01-14

**Authors:** Biqian Fu, Xiangyang Zhai, Shengyan Xi, Lifeng Yue, Yanan Wang, Yingkun Qiu, Yuewen Gong, Yangxinzi Xu, Linchao Qian, Jingru Huang, Dawei Lu, Shuqiong Huang, Jing Wang, Jing Zhou, Di Wu, Yanhui Wang

**Affiliations:** ^1^Department of Traditional Chinese Medicine, School of Medicine, Xiamen University, Xiamen 361102, Fujian, China; ^2^Cancer Research Center of Xiamen University, Xiamen 361102, Fujian, China; ^3^Dongzhimen Hospital, Beijing University of Chinese Medicine, Beijing 100700, China; ^4^School of Pharmaceutical Sciences, Xiamen University, Xiamen 361102, Fujian, China; ^5^College of Pharmacy, Rady Faculty of Health Sciences, University of Manitoba, Winnipeg R3E 0T5, Manitoba, Canada; ^6^Department of Physiology, Rady Faculty of Health Sciences, University of Manitoba, Winnipeg R3E 3P4, Manitoba, Canada; ^7^Central Laboratory, School of Medicine, Xiamen University, Xiamen 361102, Fujian, China

## Abstract

**Background:**

Ciji-Hua'ai-Baosheng II Formula (CHB-II-F) is a new traditional Chinese medical formula that has been shown to reduce toxicity and side effects of chemotherapy and increase the probability of cancer patient survival. Whether CHB-II-F is safe as an adjunctive therapy for cancer patients receiving chemotherapy has yet to be determined.

**Purpose:**

To evaluate the acute and subchronic toxic effects of CHB-II-F in rodent models.

**Methods:**

In acute toxicity test, 24 Kunming mice were divided into 2 groups: untreated control and CHB-II-F 1.05 g/mL (31.44 g/kg) treated group. Treatment was administered to the treated group 3 times a day for 14 days. The overall health, adverse reactions, and mortality rate were documented. In subchronic toxicity test, 96 Sprague-Dawley rats were divided into 4 groups: untreated control, high dose CHB-II-F (H) (26.20 g/kg), medium dose CHB-II-F (M) (13. 10 g/kg), and low dose CHB-II-F (L) (6.55 g/kg) [equal to 24.375 g (dried medicinal herb)/kg] treated groups. Treated groups were given the treatments once a day for 4 weeks. The overall health and mortality rate were recorded every day. Body weight and food consumption were measured once a week. Hematologic and biochemical parameters, organ weights, and histopathologic markers were analyzed after 4 weeks. An additional 2 weeks were given as the treatment recovery period before end-point euthanization, and biochemical analyses were performed.

**Results:**

The maximum tolerated dose (MTD) of CHB-II-F on mice was found to be 94.31 g/kg [equal to 351 g (dried medicinal herb)/kg], which is 108 times the human adult dose. In the acute toxicity test, administration of CHB-II-F 31.44 g/kg showed no adverse effect and did not cause mortality. In the subchronic toxicity test, after 4 weeks of treatment, compared to the controls, total cholesterol (TCHO) level, cardiac and splenic indexes, body weights of female rats, and mean corpuscular hemoglobin concentration (MCHC) in the CHB-II-F (H) group were significantly increased; triglyceride (TG) in the CHB-II-F (M) group and liver and splenic indexes in the CHB-II-F (L) group were increased. After the two-week recovery period, biofluid analyses, food consumption, and histopathologic examinations showed no abnormalities.

**Conclusion:**

Administration of CHB-II-F had no obvious adverse effect on the overall health of rodent models. A daily maximum dose of less than 94.31 g/kg or 6.55 g/kg CHB-II-F for 4 continuous weeks was considered safe.

## 1. Introduction

Traditional Chinese medicine (TCM), rooted in ancient Chinese medical practices, has evolved over the past thousands of years and gained popularity worldwide. In particular, Chinese herbal medicine is a branch of TCM that prescribes formulations containing naturally occurring substances to treat diseases. The application of TCM is also versatile [1, 2]. Compared to chemical synthesized or pure extracted drugs, herbs utilized in TCM are often considered to have fewer side effects when used in accordance with the principle of TCM [3], but potential adverse reactions may exist under certain contexts. For instance, improper formulation and processing of the herbal formula, as well as unwanted interactions between TCM and other medicines, can all lead to unfavorable responses [4–6]. Therefore, even though Chinese herbal medicine has been approved and used extensively in clinics, it is still necessary to evaluate its toxicology in order to ensure the highest quality and safety for usage in patients [7].

In China, the incidence and mortality rates of malignant tumor are increasing drastically. Since 2015, malignant tumor has become the leading cause of death and a major burden of health care costs [8]. At present time, chemoradiation and surgical removal of the tumor are the major therapeutic methods to treat malignant tumor in clinics. However, in addition to killing cancer cells, chemotherapy drugs are also damaging to healthy cells especially the ones that are actively dividing. According to the principle of TCM, chemotherapy drugs can induce toxicity and side effects that further decrease the body's health* qi* and blood in a cancer patient, which disharmonize the body equilibrium [9, 10]. The phenomenon has been described in* The Yellow Emperor's Inner Classic (Huang Di Nei Jing)*: “if healthy* qi* can be kept interior, pathogens cannot invade; and in order for pathogens to invade,* qi* must (first) be deficient.” To the body's healthy qi, chemotherapy side effect is considered one kind of pathogens. TCM is therefore used to strengthen health qi and eliminate pathogens, increase drug's efficiency and decrease drug's toxicity, reduce toxicity of chemotherapy, and ameliorate unwanted symptoms.

According to TCM, cancer is a malignant disease of the internal organs, four limbs, and head, which is caused by multiple factors such as deficiency of healthy qi, invasion of pathogens and toxin, depression, and disorders in drinking and eating. These factors can induce functional disorder of the internal organs, abnormal circulation of blood and body fluids, and stagnation of qi and blood. Moreover, pathogenic dampness can generate phlegm and its accumulation induces toxic heat in the viscera. All of these can contribute to tumor formation in the long run [11]. Department of Traditional Chinese Medicine of Xiang'an Hospital of Xiamen University has created a new hypothesis for cancer formation based on experience in cancer diagnosis and expertise in TCM and pointed out that tumor formation begins with the imbalance of internal environment, which causes accumulation of multiple pathological factors such as phlegm, dampness, and blood stasis. Although patients undergo surgical operation and chemoradiation therapy, the imbalance of internal environment is not corrected. Therefore, the pathological factors that are still present in the body can still induce relapse of tumor or cancer metastasis [12]. Based on this hypothesis, Xiang'an Hospital of Xiamen University proposed the Ciji-Hua'ai-Baosheng Formula (CHBF), which focuses on reinforcing the body's immunity and removing pathological factors. It has been shown to attenuate the side effects of chemotherapy and restore the balance of internal environment. Clinical observations supported the beneficial effect of CHBF on cancer patients receiving chemotherapy, which reported in a Chinese patent that CHBF was used for the treatment of dozens of lung cancer and primary liver cancer patients, and after years of observation, the results indicated that it could relieve discomfort symptoms and prolong survival time [13]. Laboratory studies have also revealed that CHBF can prolong the lifespan of mice with ascitic H_22_ hepatocellular carcinoma, inhibit tumor growth, prevent decrease of white blood cells and platelets, and improve the immune function of H_22_ tumor bearing mice receiving chemotherapy [14, 15].

Ciji-Hua'ai-Baosheng II Formula (CHB-II-F), as a new Chinese medical formula for reducing the recurrence rate of cancer patients [13], is a second generation formula refined from the original Ciji Hua'ai Baosheng Decoction (CHBD) [15] without changing the principles of treatment in order to better facilitate its subsequent applications and further development. CHB-II-F retains the most important eight medicinals in CHBD and is composed of Radix Codonopsis, Semen Ziziphi Spinosae, Fructus Hordei Germinatus, Pericarpium Citri Reticulatae, Poria, Concha Ostreae, Bulbus Fritillariae Ussuriensis, and Radix Salviae Miltiorrhizae. Radix Codonopsis can fortify the spleen and supplement the deficiency. Fructus Hordei Germinatus, Pericarpium Citri Reticulatae, and Poria can promote digestion, invigorate the stomach, move qi, strengthen the spleen, and dissolve dampness. Concha Ostreae, Bulbus Fritillariae Ussuriensis, and Radix Salviae Miltiorrhizae can soften the hardness, dissipate masses, invigorate blood, and dispel stasis. The formula is designed to remove pathogens and restore healthy* qi*, which reestablishes balance of the internal environment and decreases the recurrence rate of tumor [13].

Although CHB-II-F has been prescribed extensively in TCM clinics, its toxicity and safety have not been investigated. Therefore, the current study focuses on examining the acute and subchronic effects of CHB-II-F using mice and rats, respectively.

## 2. Materials and Methods

### 2.1. Preparation of Herbs and Decoction of Formula

CHB-II-F is composed of 8 TCM herbs as listed in Table 1. They are purchased from Yanlaifu Pharmaceutical Co., Ltd. (Xiamen, China). Each herb was identified by the experts in the Pharmacy College of Xiamen University. All voucher specimens were deposited in the Chinese Medicine Research Centre of Xiamen University for future reference. A total of 195 g of mixed CHB-II-F crude herbs were soaked in 1950 mL of water for 30 min and boiled for 30 min to yield a final volume of 200 mL. The decoction was filtered with 8 layers of surgical gauze. Herb residues were again soaked in 1500 mL water, boiled for 30 min, and filtered. Both filtered decoctions were combined and concentrated with rotary evaporation (Shanghai Yarong Biochemistry Instrument Factory, Shanghai, China) at 58°C until a final volume of 120 mL. The decoction was brought down to -80°C and lyophilized with freezer dryer (Beijing Songyuan Huaxing Technology Development Co., Ltd., Beijing, China). And the weight of final freeze-dried powder of each dose of CHB-II-F (195g) was 52.4g. The extraction yield was 26.87% and concentration of final filtered decoction was 0.44 g/mL. The lyophilized powder was sealed and stored at 4°C until use. The powder was reconstituted with distilled water to reach concentrations of 1.05 g/mL, 2.62 g/mL, 1.31 g/mL, and 0.65 g/mL for experiments.

### 2.2. UHPLC-MS

The chemical constituents of CHB-II-F extraction were profiled by ultra-high performance liquid chromatography (UHPLC) coupled with a high resolution electrospray ionization mass (HR-ESI-MS) detector. 10 mg lyophilized powder was dissolved in 1 mL of ultrapure water through ultrasonic method. The solution was filtered with 0.22 *μ*m nylon filter membrane before injection into the UHPLC. The UHPLC separation was performed over a C18Kinetex column (100×2.1 mm i.d., 2.6 *μ*m, Phenomenex Inc., Torrance, USA) on the Thermo UltiMate 3000 LC system (Thermo Fisher Scientific, Bremen, Germany). The mobile phases were acetonitrile (A) and 0.1% formic acid with water (*v/v*) (B). Samples were eluted by gradients according to the elution program as follows: A from 5% to 35% and B from 95% to 65% during 0-30 min; A from 35% to 100% and B from 65% to 0% during 30-35 min. A and B were kept at 100% and 0%, respectively, during 35 to 45 min. The column was maintained at 35°C and eluted at a flow rate of 0.3 mL/min. The injected volume was 5 *μ*L. A diode array detector with detection wavelength of 254 nm and a high resolution ESI-MS detector were used to record the HPLC chromatograms. After UHPLC, samples were analyzed by MS spectra on a Thermo Q-Exactive system. The mass spectrometer with positive and negative ionizations was calibrated across* m/z* 100-1500 using the manufacturer's calibration standards mixture (caffeine, MRFA and Ultramark 1621 in an acetonitrile-methanol-water solution containing 1% acetic acid) allowing mass fluctuation of no more than 5 ppm in the external calibration mode. The ionization voltage was 3.5 kV, and the capillary temperature was set at 300°C.

### 2.3. Experimental Animals

Twelve male and 12 female specific pathogen-free (SPF) Kunming mice at 3-4 weeks of age were used for acute toxicity test. The average weight was 20 ± 2 g. 48 male and 48 female SPF SD (Sprague-Dawley) rats at 6-8 weeks of age were used for subchronic toxicity test. The average weight was 200 ± 20 g. Both rodents were purchased from Xiamen University Laboratory Animal Center (XMULAC) in Xiamen, China [License No. SCXK (Min) 2017-0005]. Laboratory animals were kept in SPF animal house in XMULAC with routine feeding and drinking at a room temperature of 24 ± 2°C, humidity of 50 ±10%, and 12 h light-dark cycle of 7:00-19:00. Animals were given one week to adapt to the new environment before undergoing experiments. All experimental procedures were approved by the Laboratory Animal Administration and Ethics Committee of Xiamen University (No. XMULAC 2012-0039).

### 2.4. Acute Toxicity Test

According to the research guidelines in* Manufacture and Development of New Traditional Chinese Drugs* [16] and* Research Methods in Pharmacology of Chinese Materia Medica* [17], two authoritative reference books on Chinese drug development in China, maximum tolerated dose (MTD) method was employed to evaluate the acute toxicity of CHB-II-F after intragastric administration to mice. 24 mice were randomly divided into 2 groups: control and CHB-II-F treated. Each group contained 12 mice. Mice were fasted for 16 h with access to water before administration. CHB-II-F group received intragastric injection of CHB-II-F at 0.3 mL/10 g body weight at the MTD of 1.05 g/mL or 31.44 g/kg. The control group received distilled water at 0.2 mL/10 g. Intragastric administration was given 3 times a day with 6 h between each time for 14 days. For 1 h after each injection, mice were monitored for appearance, activity, respiration, secretions, defecation, and survival. Body weight was recorded once every 3 days. At the end of the study, mice were anesthetized by inhaling ethyl ether, followed by cervical dislocation. The weight and gross pathological change of liver, heart, spleen, lung, kidney, and cerebrum were documented. The indices of mouse organs were calculated according to the following equation: the indices (mg/g) = organ weight/body weight ×10.

### 2.5. Subchronic Toxicity Test

Subchronic toxicity test was carried out in accordance with the research guidelines in* Manufacture and Development of New Traditional Chinese Drugs* [16] and* Research Methods in Pharmacology of Chinese Materia Medica* [17]. 96 rats of both sexes were randomly divided into 4 groups: control and three different concentrations of CHB-II-F (26.20 g/kg, 13.10 g/kg, and 6.55 g/kg). Each group contained 24 rats. CHB-II-F groups received intragastric injection of CHB-II-F in a volume of 1 mL/100 g body weight at 2.62 g/mL, 1.31 g/mL, and 0.65 g/mL [CHB-II-F (H), CHB-II-F (M), and CHB-II-F (L), respectively] once per day for 4 weeks. The doses of the three CHB-II-F groups were equivalent to 30, 15, and 7.5 times of the clinical recommended human daily dose, respectively. Distilled water (1 mL/100 g) was used for the control group. Body weight was measured once a week and the injection volume was adjusted according to weight change. After 4 weeks, 12 rats were selected randomly and anesthetized by peritoneal injection of 10% chloral hydrate at 0.3 mL/100 g body weight. Blood was collected from the abdominal aorta. Primary organs including cerebrum, heart, liver, spleen, lung, and kidney were quickly isolated, cleaned with physiological saline, and then weighed. The relative organ weight (ROW) indices (g/g) of rats were determined by organ weight (g) / body weight (g) ×100. The other 12 rats in each group were left without further treatment for another 2 weeks to observe possible reversible toxicity reactions. The behavior and biochemical characteristics of the rats were monitored daily.

#### 2.5.1. Hematological Analysis

Blood samples were collected with either common blood collection tubes or anticoagulation tubes containing ethylene diamine tetraacetic acid (EDTA). Total white blood cell (WBC) count, WBC differential count based on lymphocyte % (Lym%), monocyte % (Mon%), neutrophil % (Neu%), eosinophil % (Eos%), and basophilic cell % (Bas%), red blood cell (RBC) count, hemoglobin (HGB), hematocrit (HCT), mean corpuscular volume (MCV), mean corpuscular hemoglobin (MCH), mean corpuscular hemoglobin concentration (MCHC), and platelet (PLT) count were measured and analyzed by BC-5500 Supermatic Hemocyte Analyzer (Shenzhen Mindray Biomedical Electronics Co. Ltd., Shenzhen, China).

#### 2.5.2. Biochemical Analysis

Blood samples in common blood collection tubes were left at room temperature for 30 min. Serum was isolated by centrifugation for 10 min at 3000 rpm at 4°C. Serum alanine aminotransferase (ALT), aspartate aminotransferase (AST), alkaline phosphatase (ALP), total protein (TP), albumin (ALB), total bilirubin (T-Bil), blood urea nitrogen (BUN), creatinine (CRE), glucose (Glu), creatine kinase (CK), total cholesterol (TCHO), triglyceride (TG), MB isoenzyme of creatine kinase (CK-MB), and gamma glutamyl transferase (GGT) were analyzed by LABOSPECT003 Supermatic Biochemistry Analyzer (Hitachi Ltd., Tokyo, Japan). Levels of sodium ion (Na^+^), potassium ion (K^+^), and chloride ion (Cl^−^) were determined by PL-1000B electrolyte analyzer (Nanjing Perlong Medical Equipment Co. Ltd., Nanjing, China).

#### 2.5.3. Urinalysis

Urine from rats was collected during the 4-week treatment and 2-week recovery periods. Leukocyte (LEU), nitrite (NIT), urobilinogen (UBG), protein (PRO), pH, occult blood, specific gravity (SG), ketone (KET), and glucose (GLU) were analyzed by U120 Pro Urine Analyzer (Hangzhou Acon Biotechnology Co. Ltd., Hangzhou, China).

#### 2.5.4. Routine Analysis of Stool

Stool samples from rats were collected during the 4-week treatment and 2-week recovery periods and used in stool saline smear. Presence of helminths, bacteria, cysts, and crystals was examined under the BL203LED Biological Microscope (Chongqing Optec Instrument Co. Ltd., Chongqing, China).

#### 2.5.5. Histopathologic Analysis

After tissues and organs were isolated and weighed, they were immediately fixed in 10% neutralized formaldehyde solution for at least 24 h and embedded in paraffin. Paraffin sections were cut in 5 *μ*m thickness and went through gradient dehydration. The sections were then stained with Hematoxylin and Eosin (H&E). Histologic changes were observed by Intellective Biological Microscope (Olympus Optical Co. Ltd., Tokyo, Japan).

### 2.6. Statistical Analysis

Parametric data were expressed as mean ± standard deviation (SD) ($\overset{-}{x}$±s). GraphPad Prism 5.0 software (GraphPad Software Inc., La Jolla, USA) was used for one-way analyses of variance (One-Way ANOVA [analysis of variance]). The least significant difference (LSD) method was chosen as the post hoc analysis. Difference of* P*<0.05 was considered statistically significant.

## 3. Results

### 3.1. Analysis of CHB-II-F by UHPLC

CHB-II-F was isolated with UHPLC system and its chromatographic fingerprinting was established (see Supplementary Material (available here)). Comparing the retention times of UV and MS spectra with reference samples, the following 10 major ingredients were identified: 3,4-dihydroxybenzaldehyde (peak 1, Rt = 3.72 min), caffeic acid (peak 2, Rt = 6.04 min), spinosin (peak 3, Rt = 12.34 min), baicalin (peak 4, Rt = 13.45 min), salvianolic acid C (peak 5, Rt = 14.10 min), hesperidin (peak 6, Rt = 15.68 min), rosmarinic acid (peak 7, Rt = 16.25 min), salvianolic acid B (peak 8, Rt = 18.89 min), lithospermic acid (peak 9, Rt = 19.42 min), and nobiletin (peak 10, Rt = 31.78 min).

### 3.2. Acute Toxicity Test Results

After successive intragastric injection of CHB-II-F for 14 days, all animals were alive in control and CHB-II-F treated groups. There were no abnormalities in appearance including hair color and gloss, behaviors and activities, food and water intakes, and excretions of these mice. As shown in Tables 2, 3, and 4, at the end of the experiments, compared to the control group, body weights, food consumption, and weights of organs in the CHB-II-F treated group were not significantly different from controls (*P*>0.05). Overall, the gross anatomy of primary organs had no abnormalities through macroscopic observation. The clinically recommended daily dosage of CHB-II-F was 3.25 g (dried medicinal herb)/kg. The maximum tolerated dose (MTD) of CHB-II-F on mice was determined by 30 mL/kg × 3 times × 1.05 g/mL [3.9 g (dried medicinal herb)/mL] = 94.31 g/kg [351 g (dried medicinal herb)/kg], which is equivalent to 108 times the adult human dose. Even at such dosage, mouse median lethal dosage (LD 50) could not be measured due to the low toxic effect of CHB-II-F. After successive administration for 14 days, mice weights, food consumption, and indices of primary organs had no statistical difference compared to those of the control group (*P*>0.05) (see Tables 2, 3, and 4).

### 3.3. Subchronic Toxicity Test Results

#### 3.3.1. Observation on General State of Health

After 4 weeks of treatment, all animals were alive. There were no abnormalities in the appearance, behavior, and activities of rats in all three CHB-II-F treated groups. No abnormal secretions were found from the eyes, ears, or genitals. Compared to the control group, color and texture of primary organs in the three CHB-II-F treated groups by macroscopic observation had no evidence of abnormalities. Weekly food intake in all three CHB-II-F treated groups also had no statistically significant difference from the controls (*P*>0.05) (see Table 5).

#### 3.3.2. Body Weight Changes

After 4 weeks of treatment, body weights of female rats in CHB-II-F (H) treated group were significantly higher than that in the control group (*P*<0.01), while body weights of other CHB-II-F treated groups had no significant differences (*P*>0.05) (see Table 6). After the two-week recovery period, body weight changes between the CHB-II-F treated groups and control group remained statistically insignificant (*P*>0.05) (see Table 7).

#### 3.3.3. Relative Organ Weight (ROW) Indices of Rats

After 4 weeks of treatment, compared to the control group, the heart and spleen indices of male rats in CHB-II-F (H) treated group were increased (*P*<0.05;* P*<0.01). In CHB-II-F (L) treated group, the spleen index of female rats was also increased (*P*<0.05), but the liver index of male rats in the same group was decreased (*P*<0.05) (see Table 8). After the two-week recovery period, the ROW indices of rats in all three CHB-II-F treated groups had no statistical differences compared to the control group (*P*>0.05) (see Table 9).

#### 3.3.4. Hematological Cytologic Analysis

After 4 weeks of treatment, compared to the control group, MCHC of female rats in CHB-II-F (H) treated group was decreased (*P*<0.05). All other hematological markers in rats treated with three different concentrations of CHB-II-F had no statistical significance (*P*>0.05) (see Table 10). After the two-week recovery period, all hematological markers in CHB-II-F treated groups compared to the controls remained statistically insignificant (*P*>0.05) (see Table 11).

#### 3.3.5. Blood Biochemical Analysis

After 4 weeks of treatment, CHO of male rats in CHB-II-F (H) treated group compared to the control group was decreased (*P*<0.05); TG of male rats in CHB-II-F (M) treated group was increased (*P*<0.05). Other blood biochemical markers showed no statistical difference compared to controls (*P*>0.05) (see Table 12). After the two-week recovery period, all blood biochemical markers in all three CHB-II-F treated groups were not statistically different from controls (*P*>0.05) (see Table 13)

#### 3.3.6. Urinalysis

After both the 4-week treatment period and 2-week recovery period, urine parameters in all three CHB-II-F treated groups had no abnormalities and no statistical differences compared to the control group (*P*>0.05) (see Tables 14 and 15).

#### 3.3.7. Routine Analysis of Stool

After both the 4-week treatment period and 2-week recovery period, the stools for routine in different groups were detected, respectively. There were no statistical differences (*P*>0.05) in the parameters analyzed of stool smears between the control group and the three CHB-II-F treated groups (see Tables 16 and 17).

#### 3.3.8. Histopathologic Analysis of Primary Organs

**Figure 1:**
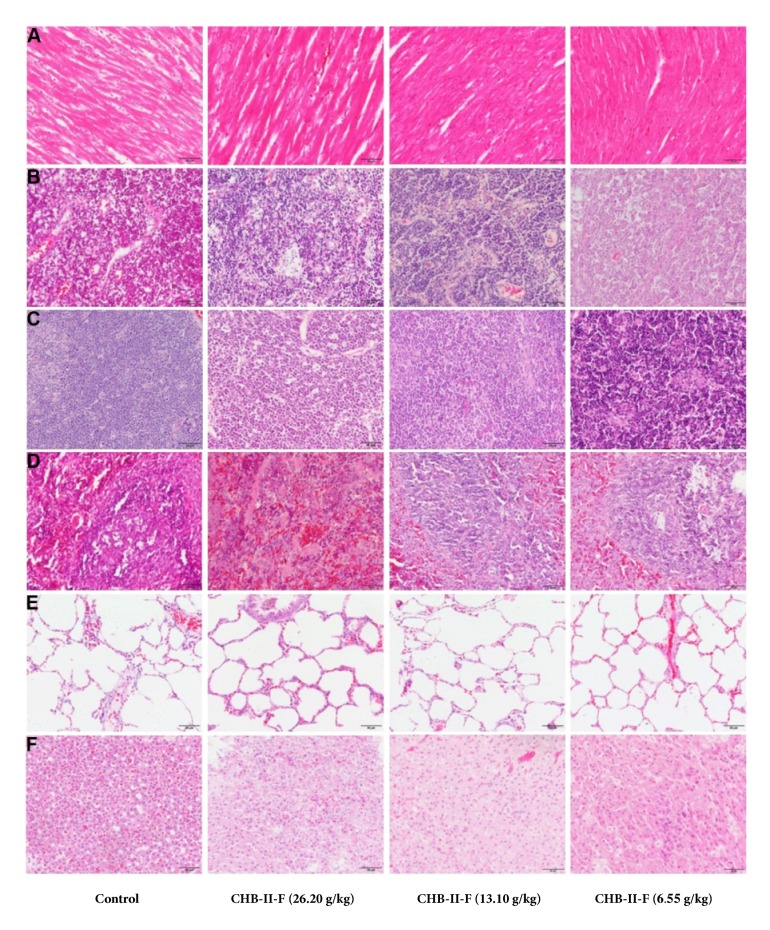
Effects of CHB-II-F on the pathology of primary organ tissues (A to F) in normal SD rats. Sections were stained with hematoxylin and eosin (H&E) and viewed at a magnification of ×400. A: heart; B: lymph gland; C: thymus; D: spleen; E: lung; F: adrenal glands.

**Figure 2:**
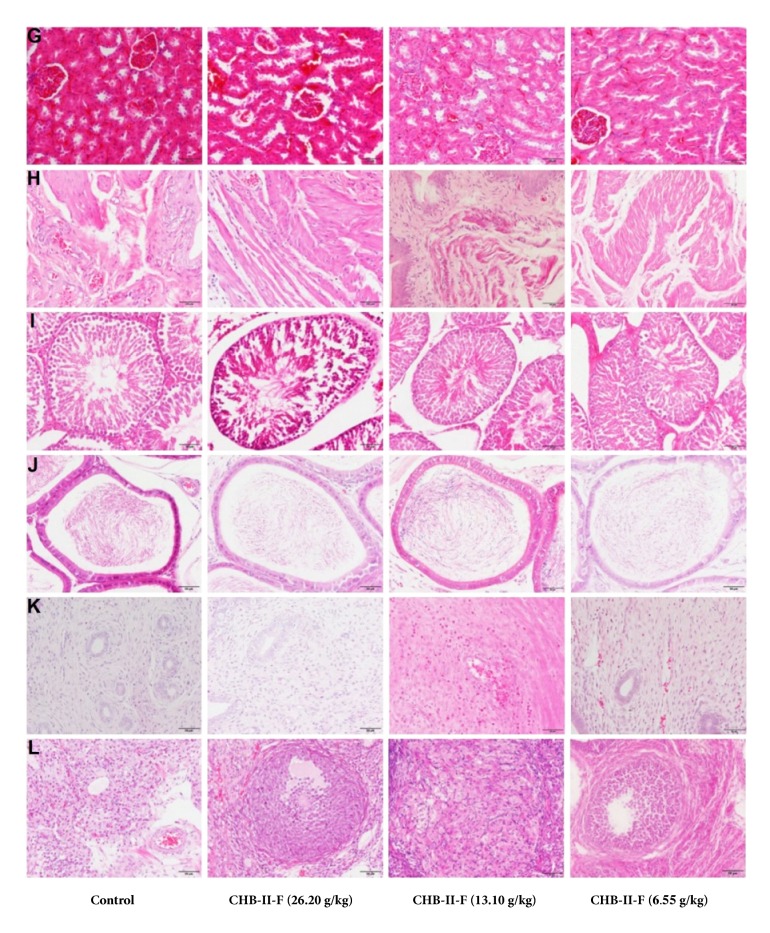
Effects of CHB-II-F on the pathology of primary organ tissues (G to L) in normal SD rats. Sections were stained with hematoxylin and eosin (H&E) and viewed at a magnification of ×400. G: kidney; H: bladder; I: testis; J: epididymis; K: ovary; L: uterus.

**Figure 3:**
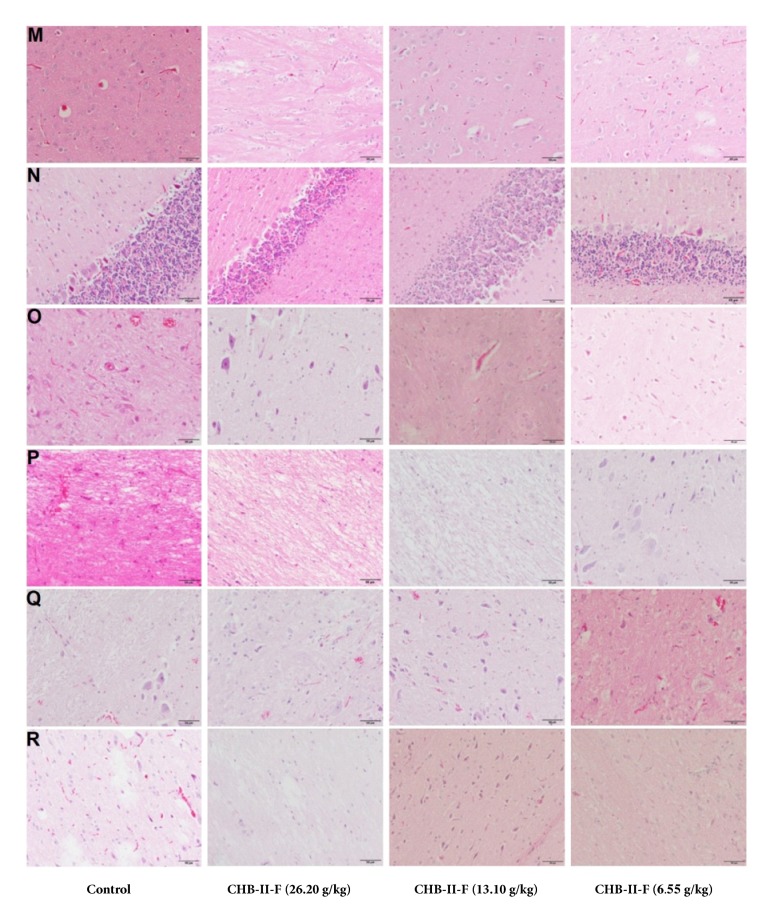
Effects of CHB-II-F on the pathology of primary organ tissues (M to R) in normal SD rats. Sections were stained with hematoxylin and eosin (H&E) and viewed at a magnification of ×400. M: brain; N: epencephal; O: brainstem; P: cervical cord; Q: thoracic cord; R: waist marrow.

**Figure 4:**
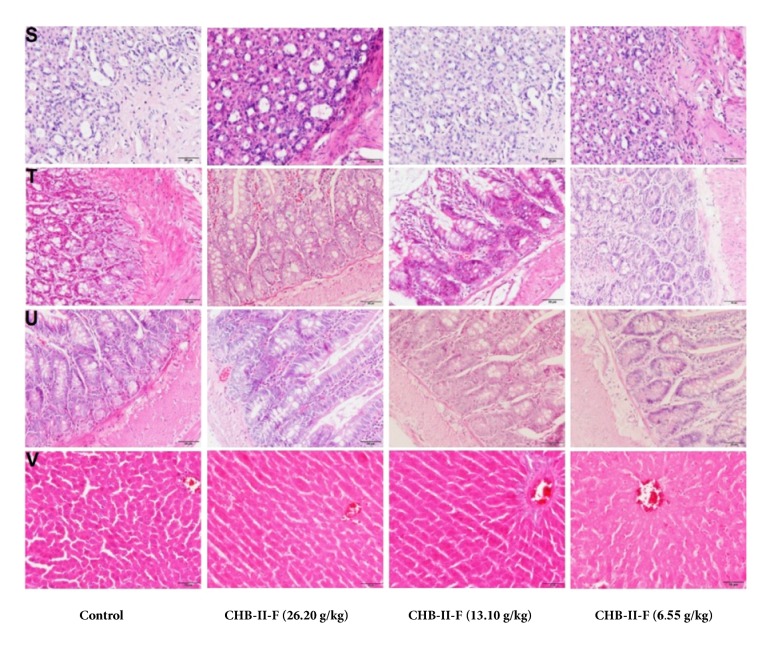
Effects of CHB-II-F on the pathology of primary organ tissues (S to V) in normal SD rats. Sections were stained with hematoxylin and eosin (H&E) and viewed at a magnification of ×400. S: stomach; T: large intestine; U: small intestine; V: liver.

To determine if CHB-II-F treatments affected the primary organ tissues of rats, histological analyses were performed. As shown in Figures 1, 2, 3, and 4, after receiving the treatment of CHB-II-F, tissue structure and morphology of the rat immune system including lymph gland, thymus, and spleen, the digestive system including stomach, large intestine, small intestine, and liver, the urinary system including kidney and bladder, the nervous system including brain, epencephala, brainstem, cervical cord, thoracic cord, and waist marrow, and the reproductive system organs including testis, epididymis, ovary, and uterus all were still normal, as well as that of heart, lung, and adrenal glands. There were no evidence of pathological changes and no abnormalities in all three CHB-II-F treated groups compared to controls.

## 4. Discussion

In certain countries around the world, traditional medicine is an integral part of the healthcare system. Medications formulated based on principles of traditional medicine are routinely prescribed to patients by healthcare practitioners. Most components in traditional medications such as those in TCM are naturally occurring and made from animals and/or plants. With the increased recognition of TCM especially Chinese materia medica (herbs) in the world, more and more patients with different health issues have accepted the treatment of Chinese materia medica and/or medical formulas [18]. Although clinical efficacy of TCM has been widely supported, its safety has not been validated by molecular analysis. Unlike allopathic medicine, toxicity and safety of TCM herbs have been based on a process of trial from early records [19]. It is therefore necessary to employ modern tools to identify potential toxicity associated with TCM herbal formulae for their successful application around the world [20].

The present study revealed that several chemical compounds such as 3,4-dihydroxybenzaldehyde, caffeic acid, baicalin, rosmarinic acid, salvianolic acid B, salvianolic acid C, and lithospermic acid were presented in CHB-II-F as determined by UHPLC. These compounds were all water-soluble components and most of them have antioxidant activities, which help remove free radicals [21–24]. 3,4-Dihydroxybenzaldehyde was reported to have vasculoprotective effects both* in vitro* and* in vivo* [25]. Spinosin may attenuate inflammation and regulate memory disorders of Alzheimer syndrome in mice [26]. Hesperidin and nobiletin were the main active components in Pericarpium Citri Reticulatae. Hesperidin is the major component of the flavonoids [27]. Nobiletin has been shown to have anticancer effects* in vitro* [28] and hesperidin has a similar effect [29]. Nobiletin, often found in citrus fruits, is an innoxious ingredient and the major component in dietary poly (methoxy) flavones. Nobiletin has been shown to induce various biological effects such as reducing inflammation and chemotherapy injury and protecting neuronal cells in mice/rats [30–32].

Based on the acute toxicity study of CHB-II-F, there was no death or adverse reactions associated with mice after intragastric injection. The maximum tolerated dose of CHB-II-F was 94.31 g/kg [equal to 351 g (dried medicinal herb)/kg], which is equivalent to 108 times of the daily recommended human adult dose [3.25 g (dried medicinal herb)/kg]. This dose far exceeds the dose used in TCM clinical application. According to the Hodge and Stemer quantifying table [33], the dose for oral CHB-II-F administration is considered safe and nontoxic.

Based on the subchronic toxicity study, there were no significant changes in the overall health, molecular markers, and survival rate of male and female rats. Changes in body and relative organ weights are used as parameters to evaluate the toxicity of drugs [34], and losing more than 10% of body weight was considered a sign of adverse reactions [35]. In the present study, after 4 weeks of CHB-II-F treatment, the average body weight of female rats in CHB-II-F (26.20 g/kg) group was significantly higher than the controls. After the two-week recovery period, body weights were not significantly different among the three CHB-II-F groups and the controls. Although a large dose of CHB-II-F orally could increase body weight, it is not considered a toxic reaction and may possibly be a good response to CHB-II-F medication. Changes in organ index mark a good indication of drug toxicity [36]. In the present study, most of the organ indices in the three CHB-II-F treated groups had no remarkable difference compared to the controls. Exceptions include an increase in the heart and spleen indices of male rats in the CHB-II-F (H) treated group and the spleen index of female rats in CHB-II-F (L) group. The liver index of male rats in CHB-II-F (L) group was decreased. Changes in organ weight may not directly reflect their functional state, but a decrease in size may signify tissue damage, which hamper drugs metabolism and its therapeutic effect [37]. When the heart is seriously damaged, there will be prominent changes in some routine biomarkers such as LDH, AST, CK, and CK-MB. These biomarkers could be employed for evaluating early cardiac toxicity [38]. Spleen is an important immune organ, and the change of spleen function is an important indicator of the body's immune system [15, 39]. The decrease of organ indices indicated that the organs were atrophic or degenerated; and increase of organ indices possibly showed that the organs were engorged, edematous, proliferative, and hypertrophic [40]. There are many factors affecting the changes of organ weights and indices of experimental animals, such as age, batch/lot, gender, and feeding season, as well as whether absolute diet before the removal of animal organs was complete, whether the removal operations were standardized, and whether the weighing was timely to avoid the evaporation of the surface water of the organs [41]. Existing reports pointed out that conceptual data of murine organ indices and biochemical indicators were close to each other on the whole but there was a slight difference, which may be related to the source and breeding environment of animal in different laboratories [42, 43]. In our present study, knowledge of the concrete medicinals and bioactive components in CHB-II-F cannot be used to explain these phenomena. Combined with the present results of blood analysis and pathological examination, the above changes of organ indices should not be directly induced by CHB-II-F, and the underlying causes need further study.

Biochemical markers in the blood provide valuable information on the effect of drug toxicity to the physiological status within the body [44]. In the subchronic toxicity test for CHB-II-F, only few biochemical markers were altered in the treatment groups compared to control. For example, in the CHB-II-F (H) group, MCHC was decreased in female rats and TCHO was decreased in male rats. In the CHB-II-F (M) group, TG was increased only in the male rats. The blood routine parameters of laboratory animals were less affected by environment and other conditions [45]. Previous research has reported that male rats had a significant higher level of MCHC compared to female rats of the same age [46], and our present study also found the same results. However, with additional statistical analysis, it was found that the MCHC change observed had no clinical significance. Blood components such as WBC, RBC, and HGB were similar compared to control. Blood biochemical values can vary based on the genotype and sex of laboratory animals [47]. Blood TG is the main biochemical indicator for internal lipid metabolism, and elevated TG is a risk factor for arteriosclerosis, coronary heart disease, and fatty liver [48]. TCHO is the sum of all cholesterols within lipoproteins in blood, which is a reflection of the lipid synthesis and reserve in the liver [49]. Although TG and TCHO were both increased in some CHB-II-F groups, it was unlikely that these changes were due to toxicity effect. According to research, TG and TCHO can fluctuate due to stress, and these changes were within the normal range of data [50]. Blood ALT and AST are the cardinal indicators of liver injury [51], and ALP is often used as an indicator of liver and gallbladder diseases, especially the obstruction of common bile duct [52]. In the present study, ALT and AST had no significant changes, and ALP was increased in female rats in the CHB-II-F (L) group, which was not considered clinically significant. In addition, all parameters measured between CHB-II-F treated groups and the controls were not significantly different after the 2-week recovery period. In summary, the observed changes during the 4-week treatment period may be due to other factors, and the specific reasons needed to be further researched and verified.

Urine samples are used to examine the health and function of the urinary system and used as supporting evidence for the diagnosis of kidney diseases [53]. Stool samples are also used to analyze pathological changes of the digestive tract based on color, microbiota, and the presence of blood. In the subchronic toxicity test, there were no abnormalities found in the urine or fecal matter of the three CHB-II-F groups. The parameters analyzed showed no significant difference compared to the control group.

The pathological changes of animal organs were evaluated macroscopically and microscopically based on guidelines from medicinal safety regulations [54]. In both the acute and subchronic toxicity tests, no pathological changes were observed in the gross anatomy. Although the organ indices of liver, spleen, and heart were changed in some CHB-II-F treated groups, analysis of tissue structures revealed no abnormalities. No pathological changes were found in the other organs. Taken together, these data strongly suggested that CHB-II-F would not induce toxicity in the body and can be safely administered to patients at regulated dosage.

Due to the complexity of Chinese medical formulas and the time constraint on the experiments, there are several limitations to the present study. First, potential bioactive components of CHB-II-F in addition to the ones identified from the results will need to be carefully characterized. Second, although drugs with a treatment course of 4 weeks [51, 55] are adequate in replicating long-term toxicity effect in Phase I clinical trials according to* Research Methods in Pharmacology of Chinese Materia Medica* [17], drug treatment of more than 6 months will be needed for subchronic toxicity test in Phases II and III clinical trials.

## 5. Conclusion

The maximum tolerated dose (MTD) of CHB-II-F was found to be 94.31 g/kg body weight [equal to 351 g (dried medicinal herb)/kg] in mice in acute toxicity test. No significant visceral pathological change was observed in rats after administration of CHB-II-F at various concentrations for 4 weeks in the subchronic toxicity test, and no adverse reactions were observed in the two-week recovery period after CHB-II-F discontinuance. A daily dose of CHB-II-F less than 94.31 g/kg body weight or 6.55 g/kg body weight administered for 4 continuous weeks was considered safe.

## Figures and Tables

**Table 1:** Contents of Ciji-Hua'ai-Baosheng II Formula (CHB-II-F).

Chinese name	Botanical name	Common name	Weight (g)	Part used	Voucher Numbers
Dang Shen	*Codonopsis pilosula* (Franch.) Nannf., *Codonopsis pilosula* Nannf. var. *modesta* (Nannf.) L. T. Shen or *Codonopsis tangshen* Oliv.	Radix Codonopsis	10	Root	160201
Fu Ling	*Poria cocos* (Schw.) Wolf	Poria	30	Sclerotium	140130
Mai Ya	*Hordeum vulgare* L.	Fructus Hordei Germinatus	20	Germinated matured fruit	131129
Chen Pi	*Citrus reticulata* Blanco	Pericarpium Citri Reticulatae	10	Matured pericarp	131129
Ping Bei Mu	*Fritillaria ussuriensis* Maxim.	Bulbus Fritillariae Ussuriensis	30	Squamous bulb	140130
Mu Li	*Ostrea gigas* Thunberg, *Ostrea talienwhanensis* Crosse or *Ostrea rivularis* Gould	Concha Ostreae	20	Shell	160201
Dan Shen	*Salvia miltiorrhiza* Bge.	Radix et Rhizoma Salviae Miltiorrhizae	50	Root and rhizome	161013
Suan Zao Ren	*Ziziphus jujuba* Mill. var. *spinosa* (Bunge) Hu ex H. F. Chou	Semen Ziziphi Spinosae	25	Matured seed	140130

**Table 2:** Body weight (g) changes of mice treated with CHB-II-F in the acute toxicity test.

	Group	Dose (g/kg)	Body weight change (g)
Male	Control	0	8.96±1.45
	CHB-II-F	31.44	10.06±1.06
Female	Control	0	5.47±0.75
	CHB-II-F	31.44	4.03±6.50

Note: Data were presented as the mean ±SD from 6 mice. No statistically significant differences were found (*P*> 0.05).

**Table 3:** Food intake dose (g) changes of mice treated with CHB-II-F in the acute toxicity test.

	Group	Dose (g/kg)	3^rd^ d	6^th^ d	9^th^ d	12^th^ d	14^th^ d
Male	Control	0	7.37±2.14	5.98±1.44	4.99±2.34	5.25±0.93	4.72±1.31
	CHB-II-F	31.44	8.21±1.98	6.94±2.19	5.93±2.99	5.61±2.75	5.43±1.06
Female	Control	0	6.55±0.12	5.19±1.33	4.12±0.63	3.90±1.51	4.72±1.78
	CHB-II-F	31.44	7.19±0.69	5.37±1.66	4.22±0.92	3.67±0.23	3.88±2.06

Note: Data were presented as the mean ±SD from 6 mice. No statistically significant differences were found (*P* > 0.05).

**Table 4:** Primary organ indices (mg/g) of mice treated with CHB-II-F in the acute toxicity test.

	Group	Dose (g/kg)	Liver	Heart	Spleen	Lung	Kidney	Brain
Male	Control	0	4.31±0.13	0.54±0.05	0.37±0.08	0.67±0.04	1.53±0.03	0.98±0.04
	CHB-II-F	31.44	4.47±0.08	0.51±0.03	0.28±0.03	0.67±0.03	1.51±0.04	0.94±0.06
Female	Control	0	4.03±0.15	0.57±0.03	0.34±0.04	0.75±0.05	1.26±0.04	1.19±0.04
	CHB-II-F	31.44	4.31±0.32	0.59±0.04	0.30±0.04	0.75±0.04	1.20±0.06	1.10±0.04

Note: Data were presented as the mean ±SD from 6 mice. No statistically significant differences were found (*P* > 0.05).

**Table 5:** Weekly Food intake dose (g) changes of rats treated with CHB-II-F in sub-chronic toxicity test.

	Group	Dose (g/kg)	Time					
			1^st^ Week	2^nd^ Week	3^rd^ Week	4^th^ Week	5^th^ Week	6^th^ Week
Male	Control	0	154.66±12.02	181.59±7.79	161.35±10.01	159.98±13.21	163.56±7.33	165.55±9.73
	CHB-II-F (H)	26.20	148.78±8.11	168.48±11.13	161.07±9.15	145.79±14.13	181.70±9.47	169.07±11.81
	CHB-II-F (M)	13.10	138.91±2.12	168.97±8.31	131.32±7.56	139.87±6.16	131.39±10.02	160.18±5.72
	CHB-II-F (L)	6.55	150.58±7.97	180.52±6.75	128.17±12.33	127.11±3.37	163.49±9.19	176.13±6.11
Female	Control	0	109.90±7.21	132.94±3.66	109.60±8.04	118.81±11.42	120.90±7.15	113.43±9.30
	CHB-II-F (H)	26.20	119.80±3.37	126.30±10.60	125.57±9.9	197.13±6.65	134.80±3.09	126.69±8.57
	CHB-II-F (M)	13.10	107.65±6.67	126.23±6.67	98.28±6.70	119.79±8.93	120.04±6.01	112.70±8.70
	CHB-II-F (L)	6.55	108.00±3.63	89.70±6.77	109.92±3.36	110.45±3.04	120.57±3.56	115.41±11.05

Note: Data were presented as the mean ±SD from 12 rats after the former 4 weeks and 6 rats after the later 2 weeks. No statistically significant differences were found (*P* > 0.05).

**Table 6:** Body weight changes (g) of rats treated with CHB-II-F after 4 weeks in the sub-chronic toxicity test.

	Group	Dose (g/kg)	Body weight changes (g)
Male	Control	0	138.97±13.86
	CHB-II-F (H)	26.20	166.08 ±8.39
	CHB-II-F (M)	13.10	111.43±10.39
	CHB-II-F (L)	6.55	153.55±6.79
Female	Control	0	58.75±3.06
	CHB-II-F (H)	26.20	87.44±7.13*∗∗*
	CHB-II-F (M)	13.10	70.72±4.96
	CHB-II-F (L)	6.55	62.98±7.35

Note: Data were presented as the mean ±SD from 12 rats. Statistical analysis: *∗P *< 0.05, *∗∗P *< 0.01 compared with control group (untreated controls).

**Table 7:** Body weight changes (g) of rats treated with CHB-II-F after 2 weeks of recovery period in the sub-chronic toxicity test.

	Group	Dose (g/kg)	Body weight changes (g)
Male	Control	0	32.11±13.86
	CHB-II-F (H)	26.20	45.20±9.00
	CHB-II-F (M)	13.10	39.49±5.96
	CHB-II-F (L)	6.55	30.29±5.26
Female	Control	0	4.85±2.88
	CHB-II-F (H)	26.20	7.25±2.76
	CHB-II-F (M)	13.10	3.88±2.55
	CHB-II-F (L)	6.55	5.19±4.86

Note: Data were presented as the mean ±SD from 6 rats. No statistically significant differences were found (*P* > 0.05).

**Table 8:** ROW indices (g/g) of rats treated with CHB-II-F for 4 weeks in the sub-chronic toxicity test.

Organ	Dose (g/kg)			
	Control	26.20	13.10	6.55
Male				
Brain	0.58±0.04	0.72±0.15	0.54±0.05	0.56±0.02
Heart	0.28±0.02	0.42±0.09*∗∗*	0.34±0.03	0.31±0.02
Liver	2.69±0.23	2.72±0.06	2.46±0.08	2.35±0.11*∗*
Spleen	0.20±0.02	0.25±0.03*∗*	0.18±0.02	0.20±0.01
Lung	0.63±0.09	0.75±0.15	0.85±0.36	0.69±0.15
Kidney	0.64±0.03	0.66±0.02	0.65±0.04	0.63±0.03
Thymus	0.13±0.01	0.18±0.04	0.16±0.04	0.15±0.03
Adrenal glands	0.02±0.02	0.02±0.01	0.02±0.03	0.02±0.01
Testes	0.50±0.04	0.51±0.03	0.53±0.05	0.46±0.03
Epididymis	0.13±0.02	0.13±0.01	0.11±0.04	0.13±0.02
bladder	0.05±0.05	0.06±0.03	0.04±0.01	0.04±0.01
Female				
Brain	0.78±0.03	0.79±0.03	0.82±0.02	0.79±0.02
Heart	0.32±0.02	0.31±0.02	0.32±0.02	0.29±0.02
Liver	2.77±0.14	2.67±0.06	2.85±0.12	2.85±0.13
Spleen	0.24±0.03	0.22±0.02	0.27±0.03	0.29±0.02*∗*
Lung	0.70±0.05	0.93±0.15	0.61±0.04	0.85±0.26
Kidney	0.67±0.51	0.62±0.03	0.63±0.01	0.63±0.02
Thymus	0.19±0.03	0.19±0.04	0.13±0.01	0.16±0.04
Adrenal glands	0.03±0.05	0.03±0.03	0.03±0.04	0.03±0.01
Uterus	0.13±0.01	0.14±0.02	0.15±0.01	0.14±0.01
Ovary	0.03±0.01	0.04±0.01	0.03±0.01	0.04±0.01
bladder	0.06±0.01	0.06±0.01	0.06±0.02	0.07±0.02

Note: Data were presented as the mean ±SD from 12 rats. Statistical analysis: *∗P *< 0.05, *∗∗P *< 0.01 compared with control group (untreated controls).

**Table 9:** ROW indices (g/g) of rats treated with CHB-II-F after 2 weeks of recovery period in the sub-chronic toxicity test.

Organ	Dose (g/kg)			
	Control	26.20	13.10	6.55
Male				
Brain	0.54±0.04	0.52±0.03	0.56±0.03	0.50±0.004
Heart	0.30±0.04	0.34±0.02	0.33±0.02	0.32±0.03
Liver	3.13±0.70	2.83±0.30	2.88±0.20	2.94±0.22
Spleen	0.23±0.05	0.19±0.06	0.21±0.06	0.20±0.03
Lung	0.61±0.15	0.62±0.12	0.55±0.24	0.54±0.19
Kidney	0.68±0.07	0.70±0.13	0.65±0.13	0.66±0.06
Thymus	0.12±0.02	0.13±0.01	0.11±0.04	0.11±0.04
Adrenal glands	0.02±0.01	0.03±0.01	0.02±0.02	0.02±0.00
Testes	0.96±0.12	0.92±0.11	1.03±0.12	0.93±0.09
Epididymis	0.30±0.09	0.29±0.06	0.35±0.04	0.33±0.05
bladder	0.04±0.01	0.03±0.01	0.04±0.01	0.03±0.00
Female				
Brain	0.78±0.04	0.76±0.13	0.73±0.08	0.73±0.16
Heart	0.35±0.03	0.33±0.03	0.33±0.02	0.32±0.02
Liver	2.85±0.15	2.90±0.24	2.78±0.26	2.91±0.26
Spleen	0.23±0.05	0.23±0.05	0.21±0.04	0.23±0.03
Lung	0.75±0.09	0.62±0.60	0.69±0.07	0.76±0.23
Kidney	0.73±0.19	0.60±0.17	0.64±0.02	0.63±0.14
Thymus	0.13±0.02	0.14±0.04	0.15±0.03	0.15±0.03
Adrenal glands	0.04±0.04	0.04±0.04	0.04±0.05	0.02±0.01
Uterus	0.27±0.09	0.25±0.06	0.28±0.09	0.33±0.10
Ovary	0.06±0.01	0.06±0.01	0.05±0.02	0.07±0.01
bladder	0.04±0.13	0.04±0.01	0.04±0.01	0.04±0.05

Note: Data were presented as the mean ±SD from 6 rats. No statistically significant differences were found (*P* > 0.05).

**Table 10:** Hematological cytologic marker values of rats treated with CHB-II-F for 4 weeks in the sub-chronic toxicity test.

Parameters	Dose (g/kg)			
	Control	26.20	13.10	6.55
Male				
WBC (10^9^/L)	4.95±0.84	5.77±0.99	4.85±0.96	4.62±1.14
Neu (%)	24.62±2.12	22.56±1.89	19.12±2.74	20.68±1.81
Lym (%)	67.06±2.25	69.50±1.05	72.50±2.74	71.36±1.79
Mon (%)	8.08±0.50	7.56±0.95	8.04±0.48	7.56±0.34
Eos (%)	0.04±0.04	0.02±0.02	0.06±0.04	0.04±0.02
Bas (%)	0.20±0.06	0.36±0.05	0.28±0.08	0.36±0.10
RBC (10^12^/L)	7.049±0.32	6.88±0.33	7.38±0.12	7.06±0.53
HGB (g/L)	149.40±5.58	146.00±7.85	161.20±3.03	154.60±5.60
HCT (%)	38.64±1.55	38.26±1.56	42.14±0.99	39.94±1.56
MCV (fL)	54.86±1.11	55.46±0.52	57.06±0.56	56.54±0.75
MCH (pg)	21.22±0.15	21.20±0.24	21.82±0.15	21.88±0.35
MCHC (g/L)	386.60±2.57	382.40±4.42	382.80±3.50	387.00±2.54
PLT (10^9^/L)	845.80±103.55	797.20±280.66	1145.40±65.12	909.00±165.48
Female				
WBC (10^9^/L)	2.03±0.55	2.70±0.42	4.60±0.91	3.75±1.22
Neu (%)	26.70±2.28	23.96±1.67	22.50±3.40	19.88±3.98
Lym (%)	65.16±2.0	67.46±1.43	69.16±3.02	70.98±3.14
Mon (%)	7.60±0.91	8.20±0.74	7.88±0.75	8.86±1.09
Eos (%)	0.08±0.06	0.02±0.02	0.02±0.02	0.04±0.04
Bas (%)	0.46±0.07	0.36±0.08	0.44±0.05	0.42±0.06
RBC (10^12^/L)	6.62±0.33	6.52±0.20	7.06±0.13	6.87±0.21
HGB (g/L)	146.00±3.53	141.80±1.52	154.60±4.52	151.40±3.51
HCT (%)	37.52±1.08	38.48±0.84	39.56±1.05	39.70±0.51
MCV (fL)	56.86±1.35	59.12±1.53	55.98±0.44	57.76±0.85
MCH (pg)	22.16±0.65	21.78±0.50	21.88±0.25	22.04±0.31
MCHC (g/L)	389.20±4.54	369.00±7.04*∗*	390.60±2.50	381.20±3.53
PLT (10^9^/L)	1088.40±40.52	1162.20±95.23	977.00±44.53	1022.60±84.33

Note: Data were presented as the mean ±SD from 12 rats. Statistical analysis: *∗P *< 0.05 compared with control group (untreated controls).

**Table 11:** Hematological cytologic marker values of rats treated with CHB-II-F after 2 weeks of recovery period in the sub-chronic toxicity test.

Parameters	Dose (g/kg)			
	Control	26.20	13.10	6.55
Male				
WBC (10^9^/L)	3.88±1.08	2.03±0.51	4.32±1.10	5.63±2.11
Neu (%)	29.60±9.01	30.60±9.36	19.88±6.23	20.50±4.09
Lym (%)	65.95±9.07	65.25±10.34	77.75±6.78	76.53±4.63
Mon (%)	2.95±0.49	2.25±0.99	1.00±0.38	2.05±0.89
Eos (%)	1.18±0.23	1.63±0.74	1.75±0.45	0.78±0.25
Bas (%)	0.33±0.06	0.28±0.03	0.20±0.04	0.25±0.03
RBC (10^12^/L)	7.81±0.15	7.55±0.56	7.98±0.22	8.16±0.24
HGB (g/L)	143.00±4.18	135.00±6.01	138.75±2.75	147.25±2.75
HCT (%)	42.25±1.06	41.50±2.69	42.53±0.88	43.68±1.10
MCV (fL)	54.13±0.70	55.13±1.02	53.35±0.95	53.53±0.32
MCH (pg)	18.38±0.30	18.00±0.59	17.40±0.29	18.03±0.21
MCHC (g/L)	339.00±3.44	326.50±8.14	326.25±1.03	336.50±2.22
PLT (10^9^/L)	995.00±30.73	915.75±56.34	1045.00±87.99	885.25±32.70
Female				
WBC (10^9^/L)	1.32±0.36	1.39±0.18	2.62±0.53	1.62±0.42
Neu (%)	21.95±4.44	32.50±6.46	17.80±5.32	17.55±1.51
Lym (%)	70.45±8.02	59.73±8.03	79.15±5.72	80.10±1.08
Mon (%)	6.23±3.55	4.30±0.66	1.75±0.13	1.63±0.64
Eos (%)	1.08±0.33	3.28±1.42	1.08±0.45	0.48±0.12
Bas (%)	0.30±0.11	0.20±0.04	0.23±0.06	0.25±0.03
RBC (10^12^/L)	7.05±0.15	7.08±0.37	6.90±0.37	7.15±0.11
HGB (g/L)	137.00±1.47	131.50±2.90	132.00±5.40	136.25±3.07
HCT (%)	39.60±0.09	39.50±1.05	39.53±0.97	40.05±0.52
MCV (fL)	56.23±1.05	56.10±1.75	57.55±1.88	55.98±0.46
MCH (pg)	19.48±0.33	18.73±0.72	19.15±0.42	19.08±0.30
MCHC (g/L)	346.00±3.19	333.50±2.75	333.50±7.37	340.00±4.74
PLT (10^9^/L)	1025.50±115.74	880.75±77.51	1065.00±84.52	857.25±92.28

Note: Data were presented as the mean ±SD from 6 rats. No statistically significant differences were found (*P* > 0.05).

**Table 12:** Blood biochemical parameters of rats treated with CHB-II-F for 4 weeks in the sub-chronic toxicity test.

Parameters	Dose (g/kg)			
	Control	26.20	13.10	6.55
Male				
ALT (U/L)	46.20±3.01	46.20±4.59	36.40±1.56	41.75±2.53
AST (U/L)	80.00±3.57	76.60±4.53	79.75±5.37	83.80±5.54
ALB (g/L)	42.12±1.20	37.58±2.24	40.50±3.56	37.72±2.20
ALP (U/L)	173.00±21.57	196.50±18.11	166.40±30.62	156.60±16.25
TP (g/L)	70.00±0.52	64.80±0.88	70.20±0.88	72.00±2.58
BUN (mmol/L)	7.06±0.65	6.36±0.50	6.18±0.51	6.93±0.50
GLU (mmol/L)	7.04±0.53	6.96±0.22	6.53±0.33	6.82±0.26
TG (mmol/L)	0.24±0.05	0.29±0.55	0.52±0.09*∗∗*	0.33±0.55
TCHO (mmol/L)	1.92±0.06	1.52±0.08*∗∗*	1.82±0.12	1.91±0.053
CK (U/L)	267.00±45.18	262.75±42.19	298.00±51.52	271.60±45.37
CRE (*μ*mmol/L)	29.46±1.55	28.86±1.52	31.32±1.60	27.80±1.49
CK-MB (U/L)	233.40±45.35	256.40±42.53	264.33±25.33	236.60±35.62
T-Bil (*μ*mmol/L)	0.41±0.15	0.04±0.02	0.06±0.05	0.08±0.03
GGT (U/L)	0.20±0.22	0.60±0.45	0.40±0.25	0.40±0.25
K (mmol/L)	4.86±0.25	5.25±0.15	5.12±0.15	5.02±0.26
Na (mmol/L)	133.56±1.56	130.96±0.35	132.34±0.46	132.02±0.75
Cl (mmol/L)	109.24±1.24	107.42±0.35	109.04±0.51	110.38±0.35
Female				
ALT (U/L)	38.00±5.23	45.40±5.34	38.80±3.51	43.40±4.56
AST (U/L)	77.20±3.52	73.60±2.15	75.00±2.54	77.75±2.53
ALB (g/L)	44.32±2.51	38.76±2.56	41.60±1.51	43.04±2.23
ALP (U/L)	87.00±8.96	94.40±8.64	66.40±4.26	113.00±16.25
TP (g/L)	75.60±5.36	68.60±0.51	72.40±1.52	76.60±2.59
BUN (mmol/L)	8.97±0.86	7.38±0.63	8.65±0.87	8.15±0.52
GLU (mmol/L)	8.39±0.76	9.02±0.26	7.95±0.51	7.53±0.75
TG (mmol/L)	0.32±0.07	0.26±0.09	0.33±0.05	0.31±0.04
TCHO (mmol/L)	1.91±0.23	1.83±0.11	1.54±0.15	1.76±0.09
CK (U/L)	188.60±15.61	196.20±17.25	165.75±8.25	147.24±9.25
CRE (*μ*mmol/L)	37.84±1.89	37.44±2.58	35.30±1.72	37.84±2.58
CK-MB (U/L)	169.80±11.28	145.40±14.26	185.80±35.26	136.75±10.38
T-Bil (*μ*mmol/L)	0.31±0.25	0.22±0.09	0.29±0.10	0.29±0.13
GGT (U/L)	0.20±0.22	0.60±0.25	0.20±0.22	0.80±0.42
K (mmol/L)	4.86±0.45	4.64±0.11	4.46±0.10	5.40±0.13
Na (mmol/L)	129.12±3.34	132.70±0.13	130.12±1.63	131.84±0.74
Cl (mmol/L)	110.04±4.60	108.56±0.68	108.40±1.02	108.24±1.52

Note: Data were presented as the mean ±SD from 12 rats. Statistical analysis: *∗∗P *< 0.01 compared with control group (untreated controls).

**Table 13:** Blood biochemical parameters of rats treated with CHB-II-F after 2 weeks of recovery period in the sub-chronic toxicity test.

Parameters	Dose (g/kg)			
	Control	26.20	13.10	6.55
Male				
ALT (U/L)	43.75±3.12	58.746±7.40	48.75±2.32	52.00±3.24
AST (U/L)	112.25±24.52	95.50±5.91	105.73±4.50	110.50±9.14
ALB (g/L)	25.13±1.54	29.75±1.54	28.93±1.52	29.58±0.55
ALP (U/L)	159.75±22.01	243.25±35.33	165.25±18.90	183.75±11.52
TP (g/L)	64.25±1.23	67.10±4.56	65.70±0.76	63.92±1.50
BUN (mmol/L)	6.12±0.51	7.27±0.24	7.16±0.23	7.39±0.65
GLU (mmol/L)	7.13±0.53	9.11±0.31	8.83±0.85	9.68±0.95
TG (mmol/L)	0.45±0.06	0.45±0.09	0.60±0.11	0.67±0.16
TCHO (mmol/L)	1.68±0.02	2.00±0.06	1.80±0.05	1.96±0.10
CK (U/L)	499.75±153.54	200.25±29.31	642.50±255.15	427.75±53.23
CRE (*μ*mmol/L)	34.50±1.55	31.53±5.25	37.85±1.35	31.03±1.52
CK-MB (U/L)	479.75±25.00	254.75±35.23	562.25±45.02	504.00±110.12
T-Bil (*μ*mmol/L)	0.78±0.19	0.55±0.19	0.93±0.22	0.95±0.14
GGT (U/L)	2.50±1.04	0.50±0.25	2.00±0.71	0.50±0.26
K (mmol/L)	5.61±0.35	9.15±0.55	6.54±0.50	6.45±0.53
Na (mmol/L)	137.20±4.45	136.65±1.14	137.63±1.56	139.96±1.54
Cl (mmol/L)	101.08±4.33	106.10±1.36	102.70±0.44	103.75±1.52
Female				
ALT (U/L)	39.00±3.37	48.00±5.42	48.50±1.52	40.00±6.45
AST (U/L)	94.00±5.25	88.50±8.60	110.25±12.36	93.50±9.52
ALB (g/L)	31.40±1.52	31.60±1.32	31.35±0.52	29.30±1.68
ALP (U/L)	79.75±5.03	102.00±14.30	93.00±3.62	88.00±13.20
TP (g/L)	70.02±3.62	66.62±2.26	69.30±1.53	64.61±3.41
BUN (mmol/L)	8.33±0.63	8.80±0.73	8.19±1.26	7.84±0.32
GLU (mmol/L)	6.58±0.36	7.07±0.23	7.36±1.02	6.69±0.62
TG (mmol/L)	0.30±0.04	0.27±0.02	0.40±0.07	0.45±0.13
TCHO (mmol/L)	1.75±0.21	1.78±0.11	2.04±0.07	2.02±0.09
CK (U/L)	401.25±94.21	176.50±16.23	379.75±81.04	326.25±61.24
CRE (*μ*mmol/L)	41.67±2.25	40.35±2.02	37.85±1.36	37.38±1.12
CK-MB (U/L)	428.00±55.36	192.50±18.04	508.75±112.23	491.50±105.41
T-Bil (*μ*mmol/L)	0.77±0.13	0.45±0.45	0.72±0.08	0.87±0.15
GGT (U/L)	1.50±0.25	0.75±0.45	1.50±0.52	1.25±0.45
K (mmol/L)	5.39±0.34	6.39±0.35	5.86±0.35	5.47±0.51
Na (mmol/L)	133.15±4.52	134.05±5.42	140.85±0.95	129.38±3.51
Cl (mmol/L)	99.15±3.50	104.50±4.35	104.49±0.16	98.58±2.53

Note: Data were presented as the mean ±SD from 6 rats. No statistically significant differences were found (*P* > 0.05).

**Table 14:** Urine parameters of rats treated with CHB-II-F for 4 weeks in the sub-chronic toxicity test.

Parameters	Dose (g/kg)			
	Control	26.20	13.10	6.55
Male				
Color	Yellow	Yellow	Yellow	Yellow
Appearance	Clear	Clear	Clear	Clear
Leucocytes	-	-	-	-
Nitrite	-	-	-	-
Urobilinogen	Normal	Normal	Normal	Normal
Protein	-	-	-	-
pH	8.67±0.33	8.67±0.17	8.00±0.76	9.00±0.00
Occult blood	-	-	-	-
Specific gravity	1.03±0.00	1.03±0.00	1.03±0.00	1.03±0.00
Ketone body	-	-	-	-
Bilirubin	-	-	-	-
Glucose	-	-	-	-
Female				
Color	Yellow	Yellow	Yellow	Yellow
Appearance	Clear	Clear	Clear	Clear
Leucocytes	-	-	-	-
Nitrite	-	-	-	-
Urobilinogen	Normal	Normal	Normal	Normal
Protein	-	-	-	-
pH	7.67±0.44	8.67±0.17	8.17±0.44	7.83±0.17
Occult blood	-	-	-	-
Specific gravity	1.02±0.00	1.03±0.00	1.03±0.00	1.03±0.00
Ketone body	-	-	-	-
Bilirubin	-	-	-	-
Glucose	-	-	-	-

Note: Data were presented as the mean ±SD from 12 rats. No statistically significant differences were found (*P* > 0.05). And “-” represents that no positive results need to be reported.

**Table 15:** Urine parameters of rats treated with CHB-II-F after 2 weeks of recovery period in the sub-chronic toxicity test.

Parameters	Dose (g/kg)			
	Control	26.20	13.10	6.55
Male				
Color	Yellow	Yellow	Yellow	Yellow
Appearance	Clear	Clear	Clear	Clear
Leucocytes	-	-	-	-
Nitrite	-	-	-	-
Urobilinogen	Normal	Normal	Normal	Normal
Protein	-	-	-	-
pH	7.72±0.02	8.03±0.70	8.01±0.65	7.31±0.11
Occult blood	-	-	-	-
Specific gravity	1.02±0.01	1.02±0.00	1.02±0.02	1.02±0.00
Ketone body	-	-	-	-
Bilirubin	-	-	-	-
Glucose	-	-	-	-
Female				
Color	Yellow	Yellow	Yellow	Yellow
Appearance	Clear	Clear	Clear	Clear
Leucocytes	-	-	-	-
Nitrite	-	-	-	-
Urobilinogen	Normal	Normal	Normal	Normal
Protein	-	-	-	-
pH	7.03±0.12	7.36±0.13	7.26±0.31	7.53±0.07
Occult blood	-	-	-	-
Specific gravity	1.01±0.00	1.02±0.00	1.03±0.00	1.02±0.00
Ketone body	-	-	-	-
Bilirubin	-	-	-	-
Glucose	-	-	-	-

Note: Data were presented as the mean ±SD from 6 rats. No statistically significant differences were found (*P* > 0.05). And “-” represents that no positive results need to be reported.

**Table 16:** Routine analysis of stool of rats treated with CHB-II-F for 4 weeks in the sub-chronic toxicity test.

Parameters	Dose (g/kg)			
	Control	26.20	13.10	6.55
Male				
Color	Yellow	Yellow	Yellow	Yellow
Character	Soft	Soft	Soft	Soft
WBC	-	-	-	-
RBC	-	-	-	-
Oil globule	-	-	-	-
Yeast	-	-	-	-
Worm eggs	-	-	-	-
Female				
Color	Yellow	Yellow	Yellow	Yellow
Character	Soft	Soft	Soft	Soft
WBC	-	-	-	-
RBC	-	-	-	-
Oil globule	-	-	-	-
Yeast	-	-	-	-
Worm eggs	-	-	-	-

Note: Data were presented as the mean ±SD from 12 rats. No statistically significant differences were found (*P* > 0.05). And “-” represents no positive results need to be reported.

**Table 17:** Routine analysis of stool of rats treated with CHB-II-F after 2 weeks of recovery period in the sub-chronic toxicity test.

Parameters	Dose (g/kg)			
	Control	26.20	13.10	6.55
Male				
Color	Yellow	Yellow	Yellow	Yellow
Character	Soft	Soft	Soft	Soft
WBC	-	-	-	-
RBC	-	-	-	-
Oil globule	-	-	-	-
Yeast	-	-	-	-
Worm eggs	-	-	-	-
Female				
Color	Yellow	Yellow	Yellow	Yellow
Character	Soft	Soft	Soft	Soft
WBC	-	-	-	-
RBC	-	-	-	-
Oil globule	-	-	-	-
Yeast	-	-	-	-
Worm eggs	-	-	-	-

Note: Data were presented as the mean ±SD from 6 rats. No statistically significant differences were found (*P* > 0.05). And “-” represents no positive results need to be reported.

## Data Availability

The data used to support the findings of this study are included within the article.

## Abbreviations

ALB: Albumin
ALP: Alkaline phosphatase
ALT: Alanine aminotransferase
AST: Aspartate aminotransferase
BUN: Blood urea nitrogen
CHB-II-F: Ciji-Hua'ai-Baosheng II Formula
CK: Creatine kinase
CK-MB: MB isoenzyme of creatine kinase
CRE: Creatinine
GGT: Gamma glutamyl transferase
GLU: Glucose
HCT: Hematocrit
HE: Hematoxylin and eosin
HGB: Hemoglobin
KET: Ketone
LEU: Leukocyte
MCH: Mean corpuscular hemoglobin
MCHC: Mean corpuscular hemoglobin concentration
MCV: Mean corpuscular volume
NIT: Nitrite
PLT: Platelet
PRO: Protein
RBC: Red blood cell
SG: Specific gravity
T-Bil: Total bilirubin
TCHO: Total cholesterol
TCM: Traditional Chinese medicine
TG: Triglyceride
TP: Total protein
UBG: Urobilinogen
UHPLC: Ultra-high performance liquid chromatography
WBC: White blood cell.

## Supplementary Materials

Supplementary Materials Supplementary Fig. A: UHPLC-MS fingerprint chromatogram of CHB-II-F. B: Chemical structure of the identified active ingredients of CHB-II-F. 3,4-Dihydroxybenzaldehyde (1), caffeic acid (2), spinosin (3), baicalin (4), salvianolic acid C (5), hesperidin (6), rosmarinic acid (7), salvianolic acid B (8), lithospermic acid (9), and nobiletin (10).
