# Supplementary material for: Safety Evaluation of a New Traditional Chinese Medical Formula, Ciji-Hua'ai-Baosheng II Formula, in Adult Rodent Models
Source: Evid Based Complement Alternat Med. 2019 Jan 14;2019:3659890. doi: 10.1155/2019/3659890 (PMC6348800; doi:10.1155/2019/3659890)
Supplement: Supplementary Materials — Supplementary Fig. A: UHPLC-MS fingerprint chromatogram of CHB-II-F. B: Chemical structure of the identified active ingredients of CHB-II-F. 3,4-Dihydroxybenzaldehyde (1), caffeic acid (2), spinosin (3), baicalin (4), salvianolic acid C (5), hesperidin (6), rosmarinic acid (7), salvianolic acid B (8), lithospermic acid (9), and nobiletin (10). [file 3659890.f1.docx]

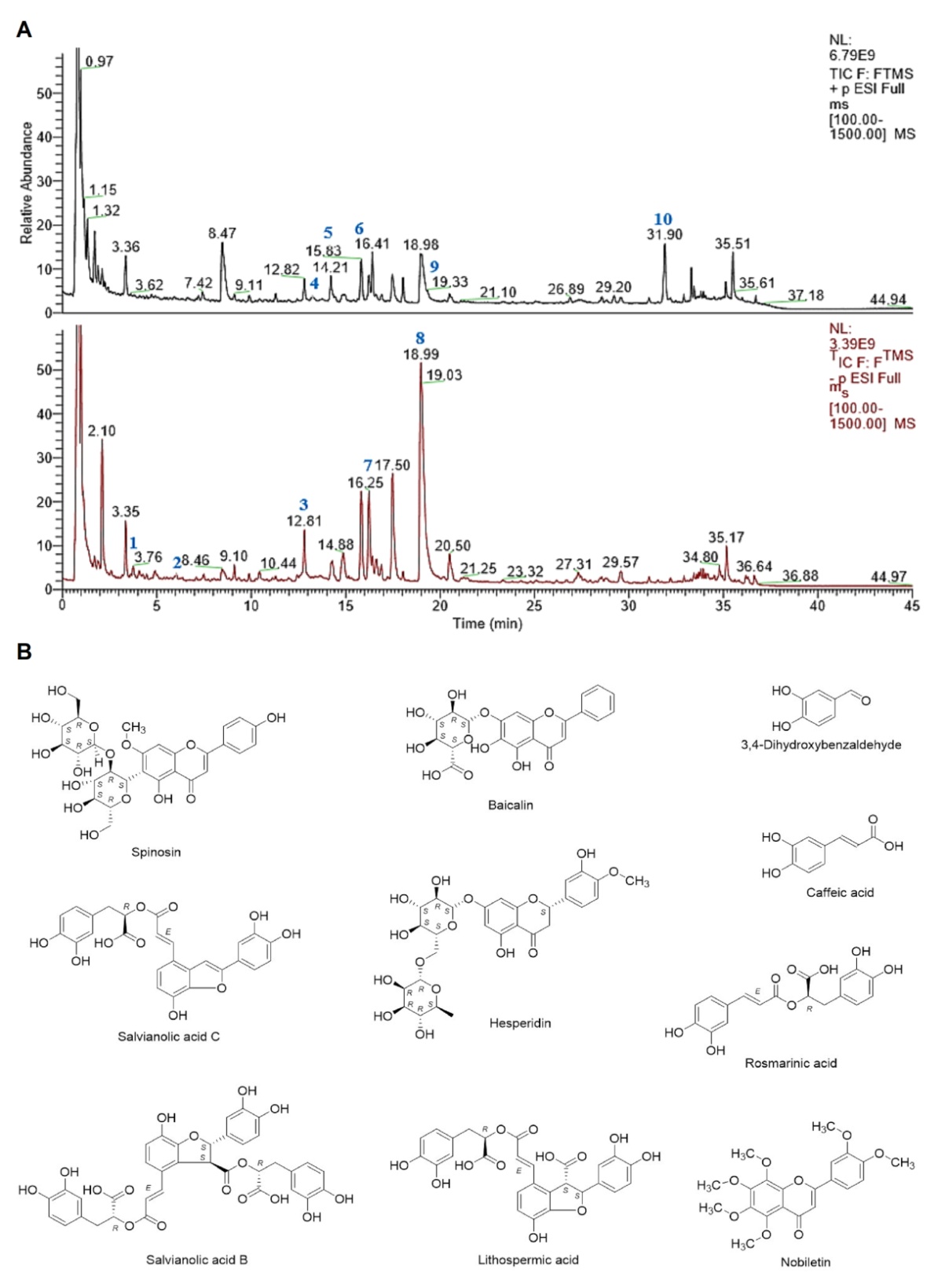


**Supplementary Fig. A:** UHPLC-MS fingerprint chromatogram of CHB-II-F. **B:** Chemical structure of the identified active ingredients of CHB-II-F. 3,4-Dihydroxybenzaldehyde (**1**), caffeic acid (**2**), spinosin (**3**), baicalin (**4**), salvianolic acid C (**5**), hesperidin (**6**), rosmarinic acid (**7**), salvianolic acid B (**8**), lithospermic acid (**9**) and nobiletin (**10**).
